# A new DNA aptamer which binds to SARS-CoV-2 spike protein and reduces pro-inflammatory response

**DOI:** 10.1038/s41598-024-58315-0

**Published:** 2024-03-29

**Authors:** Woong Kim, Eun Su Song, Song Ha Lee, Seung Ho Yang, Junhyung Cho, Seok-Jun Kim

**Affiliations:** 1 Institute of Well-Aging Medicare & Chosun University LAMP Center, Chosun University, Gwangju, 61452 Republic of Korea; 2 Corporate Research Institute, UNICOMPANY, Gwangju, 61008 Republic of Korea; 3 Department of Business Management, UNICOMPANY, Gwangju, 61008 Republic of Korea; 4 Department of Planning Management, UNICOMPANY, Gwangju, 61008 Republic of Korea; 5 Division of Emerging Viral Diseases and Vector Research, Centre for Infectious Diseases Research, Korea National Institute of Health, Korea Centres for Disease Control and Prevention Agency, Cheongju, 28159 Republic of Korea; 6 Department of Integrative Biological Sciences & BK21 FOUR Educational Research Group for Age-Associated Disorder Control Technology, Chosun University, Gwangju, 61452 Republic of Korea; 7 Department of Biomedical Science, Chosun University, Gwangju, 61452 Republic of Korea

**Keywords:** Aptamer, SARS-CoV-2, ACE2, Prevention, Diagnostic, Drug discovery, Molecular biology, Molecular medicine

## Abstract

COVID-19 caused by SARS-CoV-2 spread rapidly around the world, endangering the health of people globally. The SARS-CoV-2 spike protein initiates entry into target cells by binding to human angiotensin-converting enzyme 2 (ACE2). In this study, we developed DNA aptamers that specifically bind to the SARS-CoV-2 spike protein, thereby inhibiting its binding to ACE2. DNA aptamers are small nucleic acid fragments with random structures that selectively bind to various target molecules. We identified nine aptamers targeting the SARS-CoV-2 spike protein using the systematic evolution of ligands by exponential enrichment (SELEX) method and selected three optimal aptamers by comparing their binding affinities. Additionally, we confirmed that the DNA aptamers suppressed pro-inflammatory cytokines induced by the SARS-CoV-2 spike protein in ACE2-overexpressing HEK293 cells. Overall, the DNA aptamer developed in this study has the potential to bind to the SARS-CoV-2 spike protein and inhibit or block its interaction with ACE2. Thus, our DNA aptamers can be used as new biological tools for the prevention and diagnosis of SARS-CoV-2 infection.

## Introduction

Severe acute respiratory syndrome coronavirus-2 (SARS-CoV-2), responsible for the coronavirus disease (COVID-19) pandemic in 2019, has spread rapidly worldwide and has caused respiratory illnesses in large populations^1,2^. Viral infections can cause various adverse effects, including respiratory complications. Increased local inflammation induces excessive cytokine production or a cytokine storm, which leads to a systemic inflammatory response syndrome^3–5^. SARS-CoV-2 is a single-stranded RNA virus comprising a spike, membrane, envelope, and nucleocapsid^3^. The extracellular domain spike protein plays a key role in receptor recognition and cell membrane fusion, and contains a receptor-binding domain (RBD) that binds to the angiotensin-converting enzyme 2 (ACE2) receptor in humans.. The RBD of the S1 subunit of SARS-CoV-2 binds ACE2 and initiates its entry into target cells^6^.

The characteristics of SARS-CoV-2 are closely associated with its detection, prevention, and treatment. Early detection of SARS-CoV-2 is essential for its rapid isolation and contact tracing to reduce the risk of transmission^7^. Clinical detection, a common detection method for SARS-CoV-2 based on clinical symptoms and contact history with other potentially infected individuals, resulting in unreliable results in asymptomatic and inaccurate contact histories. Therefore, various molecular-technology-based diagnostic tests for SARS-CoV-2 have been developed. Two representative detection methods are viral nucleic acid detection methods such as reverse transcription polymerase chain reaction (RT-PCR) and quantitative real-time PCR (qPCR)^8,9^, and an immunoassay using SARS-CoV-2-specific IgM/IgG^10–12^. mRNA vaccines Pfizer–BioNTech (BNT162b2)^13^ and Moderna COVID-19 (mRNA-1273)^14^ are currently being used to prevent the spread of SARS-CoV-2. mRNA vaccines show high efficacy in preventing SARS-CoV-2 infection, but have disadvantages such as the need for a booster shot, difficulty in storage and transport, and unknown side effects. Treatment strategies for SARS-CoV-2 infection are being researched, including antiviral drugs (*remdesivir, favilavir*), anti-HIV drugs (*lopinavir, ritonavir*), and ACE2 inhibitors (*APN01*)^15,16^. Despite research efforts to treat SARS-CoV-2 infections, an effective treatment strategy has not yet been developed. Various strategies have been proposed for the prevention, detection, and treatment of SARS-CoV-2 infection, and on-going research is being based on the direct spike protein–ACE2 interaction with a focus on development of biomolecules, such as antibodies^17,18^ and aptamers^19–21^, that block this binding, preventing entry of the virus.

Aptamers are small nucleic acid fragments (DNA or RNA) with stable 3D structures that can bind to various target molecules with high affinity and selectivity^22^. Aptamers have several advantages and are used in various fields. In particular, compared to antibodies, they have advantages such as cost-effectiveness, smaller size, and lower immunogenicity^23^. Systematic evolution of ligands by exponential enrichment (SELEX) refers to the process of selecting an aptamer that binds to a specific target through an iterative selection process from a random sequence pool^24^. These aptamers are distinct from other nucleic acid-based therapies because they often have no direct effect on the steps prior to the protein function^25^. No significant differences exist in the affinities of DNA and RNA aptamers; however, DNA aptamers are more stable and therefore greatly applicable in many fields^26^.

In this study, we identified aptamers targeting SARS-CoV-2 spike proteins using the SELEX method. Nine aptamer candidates were screened using SELEX. Among these, three optimal aptamers were selected based on their binding affinities, and their superiority was confirmed by comparison with previously developed aptamers. In addition, the secondary structures of the three selected aptamers were predicted to confirm their structural characteristics. Finally, the selected aptamers were tested with the SARS-CoV-2 spike protein in ACE2-overexpressing Human embryonic kidney 293 (HEK293) cells, and the pro-inflammatory response was examined.

## Materials and methods

### SELEX procedures

We performed an aptamer selection procedure for the SARS-CoV-2 spike protein using our modified SELEX method. The single-stranded DNA (ssDNA) library was constructed from two regions with PCR primers on either side, and a region with 40 random sequences in the middle (5′-ATG CGA ATT CAT CAG TGC CAG TCA T—N40—GAT TAG CAT AGA TGA GGA TCC ATG C -3′). First, the ssDNA library was annealed at 95 ℃ and immediately cooled on ice for 10 min. The DNA aptamer that was non-specifically bound to the nickel–nitrilotriacetic acid (Ni–NTA) magnetic nanobeads was removed through a pre-cleaning step. To select DNA aptamers that specifically bind to the recombinant human coronavirus SARS-CoV-2 spike glycoprotein S1 (His tag; ab273067; Abcam, UK), the interaction between pre-cleaned DNA aptamers and spike proteins was mediated at 4 °C on a rotator for 30 min. The spike protein became bound to the DNA aptamer that had specifically bound to the Ni–NTA magnetic nanobeads (Bioneer, Daejeon, Korea) in a binding buffer containing 50 mM sodium phosphate, 500 mM NaCl, and 10 mM imidazole, pH 8.0. The aptamer-protein-nanobead conjugate was now separated using a magnet, and the supernatant was removed. The recovered beads were then washed five times with binding buffer. After washing, the DNA aptamer-protein component was released from the nanobeads in the conjugate using an elution buffer containing 50 mM sodium phosphate, 500 mM NaCl, and 500 mM imidazole, pH 8.0. The recovered DNA aptamer-protein was added to the PCR mixture TOPsimple™ PCR DyeMIX-nTaq, which included nTaq (0.2 unit/μL), nTaq buffer with 3 mM MgCl_2_, dNTP mixture (0.4 mM each), stabilizer, and dyes (Xylene cyanol and Orange G) from Enzynomics (Daejeon, Korea). The mixture was amplified using forward (5′- ATG CGA ATT CAT CAG TGC CAG TCA T-3′) and reverse (5′- GAT TAG CAT AGA TGA GGA TCC ATG C-3′) primers to amplify the target-bound sequences. PCR steps of denaturation, annealing, and extension were cycled 25 times to amplify the target DNA. The resulting PCR product, approximately 100 bp in size, was separated by agarose gel electrophoresis and purified using an Expin Combo GP Mini Kit from GeneAll (Seoul, Korea). After elution of the double-stranded DNA, it was incubated with 5 U of lambda exonuclease from Enzynomics (Daejeon, Korea) in a total reaction volume of 20 µL at 37 °C. The reaction was terminated after 30 min by incubating at 75 °C for 10 min. The resulting ssDNA product was used in the next round of the selection process, and this process was repeated four times, with the amount of protein (1, 0.5, 0.25, 0.1 µg) decreasing at each step.

### Cloning and sequencing aptamers

The pGEM®-T Easy Vector System (Promega, CA, USA) was used to clone the various aptamers selected through SELEX. The pGEM®-T Easy Vector was provided in linear form with a single 3′-T overhang for TA cloning. The selected aptamers were amplified with the addition of adenine (A) at the 3′-termini using Ex Taq™ (Takara, Shiga, Japan), and the PCR products were used for cloning. The PCR products and pGEM®-T Easy Vector were ligated at 4 °C overnight using T4 DNA ligase. Next, aptamers + pGEM®-T Easy Vector constructs were transformed into HIT Competent Cells™-DH5α (RBC, Toronto, Canada), and the resulting colonies were screened by ampicillin. The plasmid DNA was purified using the FavorPrep™ Plasmid Extraction Mini Kit (FAVORGEN, Taipei City, Taiwan). Plasmid DNA was sequenced by Sanger sequencing (Macrogen, Seoul, Korea).

### Comparison of binding affinity

To measure the binding efficiency of aptamers, 2 μM of candidate aptamers were mixed with 0.5 μg/mL of the spike protein, followed by incubation at 4 °C for 30 min. The spike protein bound to the DNA aptamers was incubated with Ni–NTA magnetic nanobeads and the conjugate was separated using a magnet, with the supernatant removed. The recovered beads were then washed five times with a binding buffer, and the aptamers were released from the nanobeads using an elution buffer. The candidate aptamers were detected using TOPreal™ SYBR Green qPCR PreMix (Enzynomics, Daejeon, Korea) to obtain the standard curve for real-time PCR analysis. Cycle threshold (Ct) values of the candidate aptamers were compared using a Rotor-Gene 3000 real-time PCR system (Corbett Research, Australia). All analyses were performed in triplicate and Ct values were calculated from the average of each measurement.

### Secondary structure prediction

The RNAfold web server (http://rna.tbi.univie.ac.at)^27^ was used to predict the secondary structures of DNA aptamers. RNAfold can be used to predict the secondary structures of single-stranded RNA or DNA sequences. It uses a partition function for sequences of up to 7,500 nt and a minimum free energy algorithm for sequences of up to 10,000 nt to predict the secondary structures.

### Cell culture and transfections

HEK293 cells were purchased from the Korea Cell Line Bank (KCLB, Seoul, Korea). The cell lines were cultured in a Dulbecco's Modified Eagle Medium (DMEM) (WELGENE Inc., Gyeongsan-si, Korea) supplemented with 10% fetal bovine serum (Corning Costar, NY, USA) and 1% penicillin/streptomycin (Gibco, NY, USA) at 37 °C in a 5% CO_2_ atmosphere. ACE2 overexpression in HEK293 cells was induced using pcDNA3.1-ACE2. The human ACE2 plasmid was obtained from Addgene (#154962)^28^. Transfections were carried out using Lipofectamine 2000 reagent (Invitrogen, Carlsbad, CA, USA) according to the manufacturer's instructions.

### RNA isolation, cDNA synthesis, and qPCR

Total RNA was isolated from HEK293 cells using RNAiso Plus reagent (Takara, Shiga, Japan), and cDNA was synthesized using ReverTra Ace™ qPCR RT Master Mix (TOYOBO, Osaka, Japan) according to the manufacturer's protocols. The expression level of IL-1β, IL-6, and TNF-α was measured in Rotor-Gene 3000 real-time PCR system (Corbett Research, Australia) using TOPreal™ SYBR Green qPCR PreMIX (Enzynomics, Daejeon, Korea).

### Western blotting

HEK293 cells were lysed using RIPA buffer supplemented with a protease inhibitor cocktail (GenDEPOT, TX, USA). The prepared proteins were separated by sodium dodecyl sulfate–polyacrylamide gel electrophoresis and electrotransferred to PVDF membrane (Millipore, MA, USA). The blocking step was carried out using 5% skim milk and washed three times with washing buffer (1X PBS and 0.05% Tween 20). After washing, the membrane was incubated over-night at 4 °C with ACE2 (sc-390851, SCBT, CA, USA) and GAPDH antibodies (AP0066, Bioworld Technology, MN, USA), and secondary antibodies were incubated for 2 h at room temperature. Finally, protein expression levels were detected using ECL solution (Bio-Rad, Hercules, CA, USA).

### ELISA

An ELISA test was used to analyze culture medium cytokine (IL-1β, IL-6, and TNF-α) levels in HEK293 cells with SARS-CoV-2 spike glycoprotein S1 and aptamer. HEK293 cells overexpressing ACE2 were treated with SARS-CoV-2 spike glycoprotein S1 and aptamers for 24 h. After incubation, the culture medium was collected and immediately frozen for further analysis. The concentrations of IL-1β, IL-6, and TNF-α in culture medium were measured using ELISA kit (Invitrogen, MA, USA) in accordance with the manufacturer's protocol.

### Statistical analysis

*P*-values < 0.05 were considered to be statistically significant. Statistical analyses were performed using GraphPad Prism version 8. Statistical differences between groups were analyzed using Student’s *t*-test. (*, **, ***, and ns indicate *P* < 0.1, *P* < 0.01, *P* < 0.001, and not significant, respectively).

## Results

### Selection and identification of DNA aptamers against the SARS-CoV-2 spike protein

**Figure 1:**
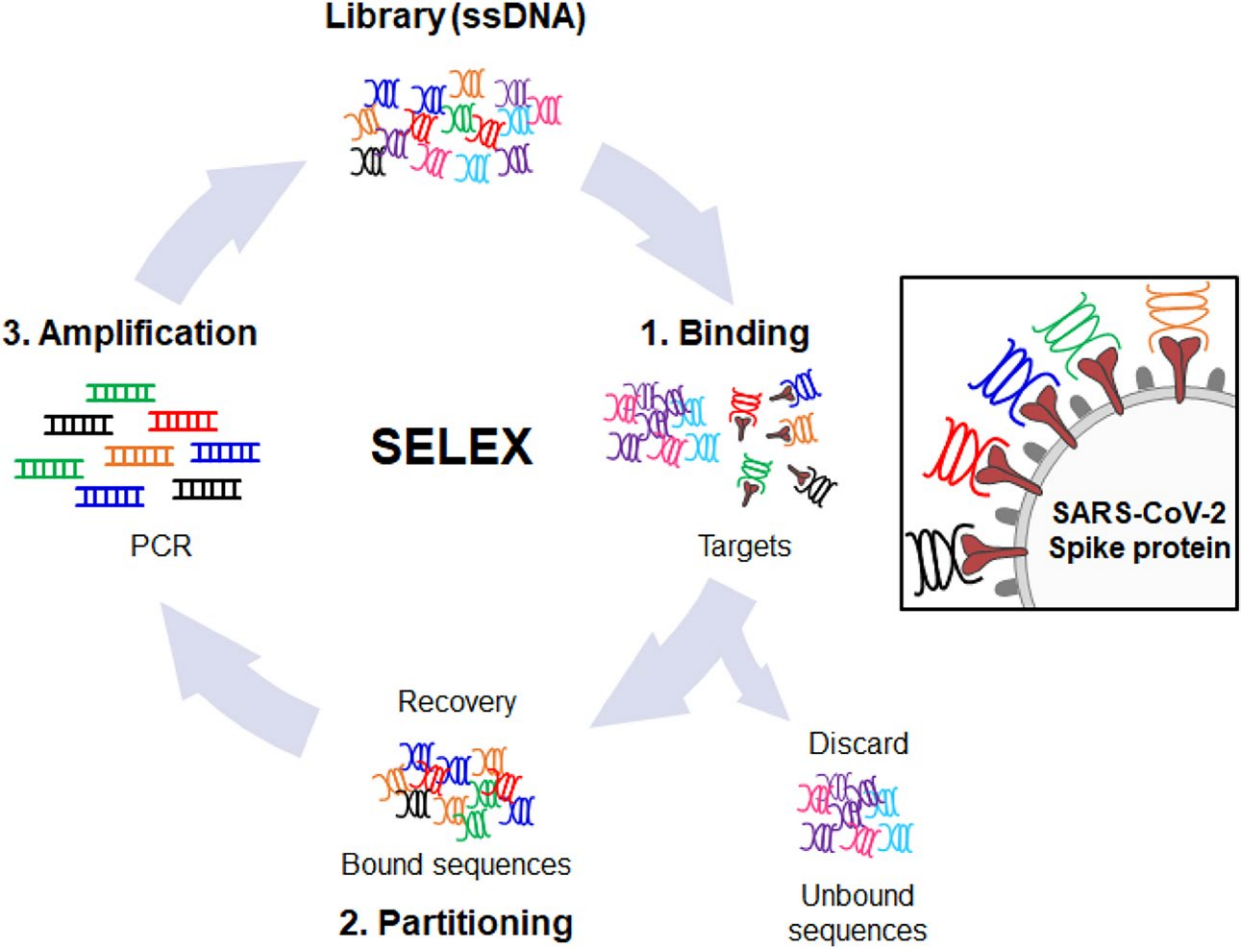
Schematic description of SELEX in the selection against the SARS-CoV-2 spike protein. Initially, a pre-clearing step was performed by incubating single-stranded DNA (ssDNA) libraries with Ni–NTA nanobeads to remove Ni–NTA binding ssDNA. The remaining unbound ssDNA was then mixed with SARS-CoV-2 spike proteins for the selection process. The binders, which specifically bound to the spike proteins, were subsequently eluted and amplified using PCR. This process was repeated four times to enhance the binding affinity of the aptamers. Finally, the DNA aptamer with improved binding affinity was cloned and its sequence was determined through sequencing.

SARS-CoV-2 spike protein-specific aptamers were selected using the SELEX method^29^. A modified SELEX method was used to identify single-stranded DNA aptamers that could bind to the SARS-CoV-2 spike protein and inhibit its binding to the ACE2 receptor. The selection process of specific ssDNA aptamers for the SARS-CoV-2 spike protein leveraged the properties of the Ni–NTA nanobeads. Initially, the ssDNA aptamer was bound to the His-tagged SARS-CoV-2 spike protein, and this binding complex was subjected to a selection process using Ni–NTA nanobeads capable of binding to the His-tagged protein. The selected aptamers were amplified by PCR, and the SELEX process was repeated four times to enhance the binding affinity of the aptamers (Fig. 1). After each selection round, the DNA aptamers were amplified via PCR and subsequently cloned into chemically competent E. coli using the pGEM®-T Easy Vector Systems. The sequences of the nine individual aptamer clones were determined (Table 1). These nine new sequences were previously unexplored; therefore, subsequent experiments were performed using these sequences.

**Table 1:** List of DNA aptamers with insert sequences.

No	Name	5′-Oligo Seq-3′
1	SC2_R4_1	ATG CGA ATT CAT CAG TGC CAG TCA **TTT CCG GTG GTC TTC GCA TAC GGC TTG ATT TGA TGC G**GA TTA GCA TAG ATG AGG ATC CAT GC
2	SC2_R4_3	GCA TGG ATC CTC ATC TAT GCT AAT C**CG TAG ACG CGT GAG GCA GCC GTA TGC GAA TCG GCA CTC CCA AC**G ATG ACT GGC ACT GAT GAA TTC GCA T
3	SC2_R4_4	GCA TGG ATC CTC ATC TAT GCT AAT C**GT TGA CAC CAG CAT ATG CGA ATC GGA GAT AAG AG**G ATG ACT GGC ACT GAT GAA TTC GCA T
4	SC2_R4_5	ATG CGA ATT CAT CAG TGC CAG TCA T**TT GCG GGT GTG TCG CTC GTC TTC GCA TAG GTG GCG CGT TG**G ATT AGC ATA GAT GAG GAT CCA TGC
5	SC2_R4_8	GCA TGG ATC CTC ATC TAT GCT AAT C**AC GAC CCA TAC GTC GTG GCT AGT ATG CGA AGA CCC CAA** GAT GAC TGG CAC TGA TGA ATT CGC AT
6	SC2_R4_10	GCA TGG ATC CTC ATC TAT GCT AAT C**CC AAT GAC GCG GAC GTA TGC GAA GGC GGA TCA AAT A**GA TGA CTG GCA CTG ATG AAT TCG CAT
7	SC2_R4_13	ATG CGA ATT CAA TCA GTG CCA GTC AT**T TGC GGG TGT GTC GCT CGT CTT CGC ATA GGT GGC GCG TTG** GAT TAG CAT AGA TGA GGA TCC ATG C
8	SC2_R4_14	ATG CGA ATT CAT CAG TGC CAG TCA T**TC TCG GGG TGC GTA TTC GCA TAA GCC CCA TCT TCA GAC CA**G ATT AGC ATA GAT GAG GAT CCA TGC
9	SC2_R4_16	ATG CGA ATT CAT CAG TGC CAG TCA T**GC GTT TCC GGT CTT CGC ATA CGT TCT CTG TCC TGG CTT** GAT TAG CAT AGA TGA GGA TCC ATG C

### Comparison of binding affinity between candidate DNA aptamers

**Figure 2:**
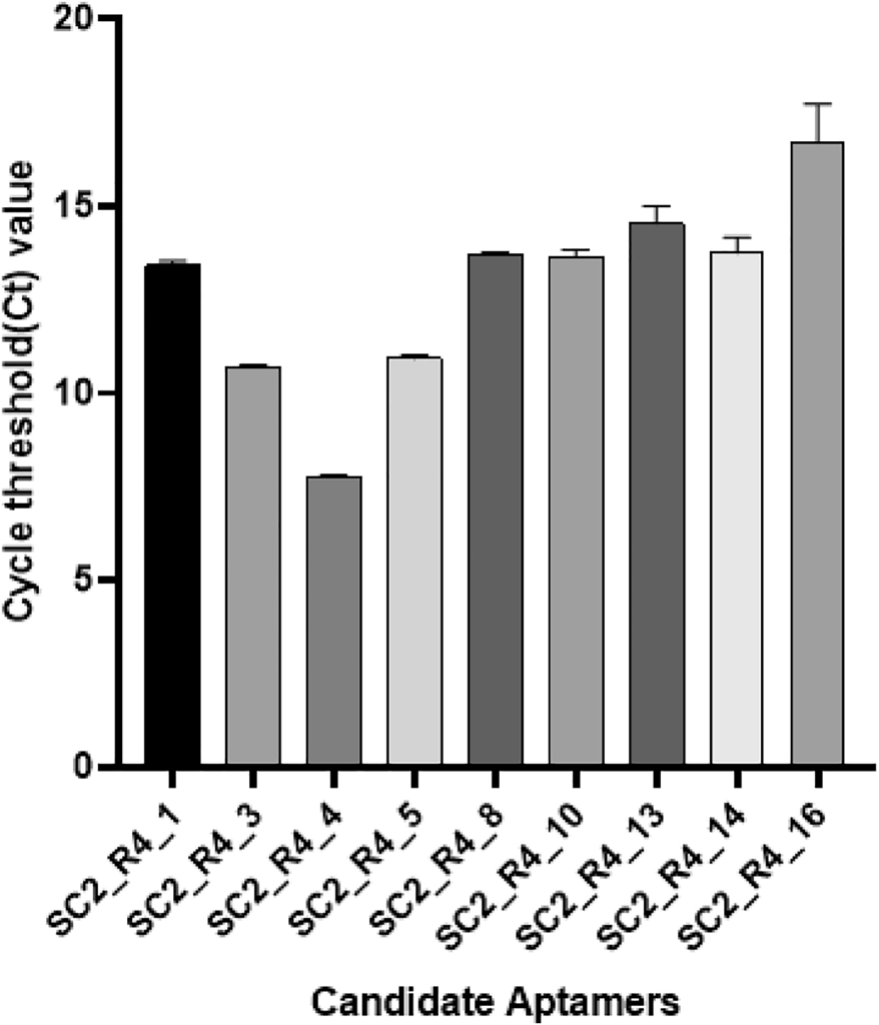
Comparison of cycle threshold (Ct) values between aptamers. The Y-axis represents the Ct values, and each bar on the X-axis corresponds to candidate DNA aptamer. The Ct value serves as an indicator of the DNA aptamer concentrations during the SELEX process under identical conditions. Error bars indicate the standard deviations obtained from three parallel tests.

To evaluate the binding affinity of the SARS-CoV-2 spike protein to the candidate DNA aptamers, qPCR analysis was conducted. When the binding of the target protein and DNA aptamer was induced under the same conditions, the aptamer with higher affinity showed more binding, and these aptamers were harvested again and quantified by qPCR. Put differently, the lower the Ct value (high concentration of DNA aptamer), the higher the binding affinity of the DNA aptamer to its target^30–33^. Therefore, by monitoring the change in Ct values using qPCR, the binding affinity of the protein target can be determined. The Ct values of the candidate DNA aptamers are shown in Fig. 2. Among them, aptamer candidates SC2_R4_3 (10.71 ± 0.05), SC2_R4_4 (7.78 ± 0.04), and SC2_R4_5 (10.93 ± 0.09) exhibited the highest binding affinities. Notably, SC2_R4_4 displayed the highest binding affinity among the three aptamer candidates.

**Figure 3:**
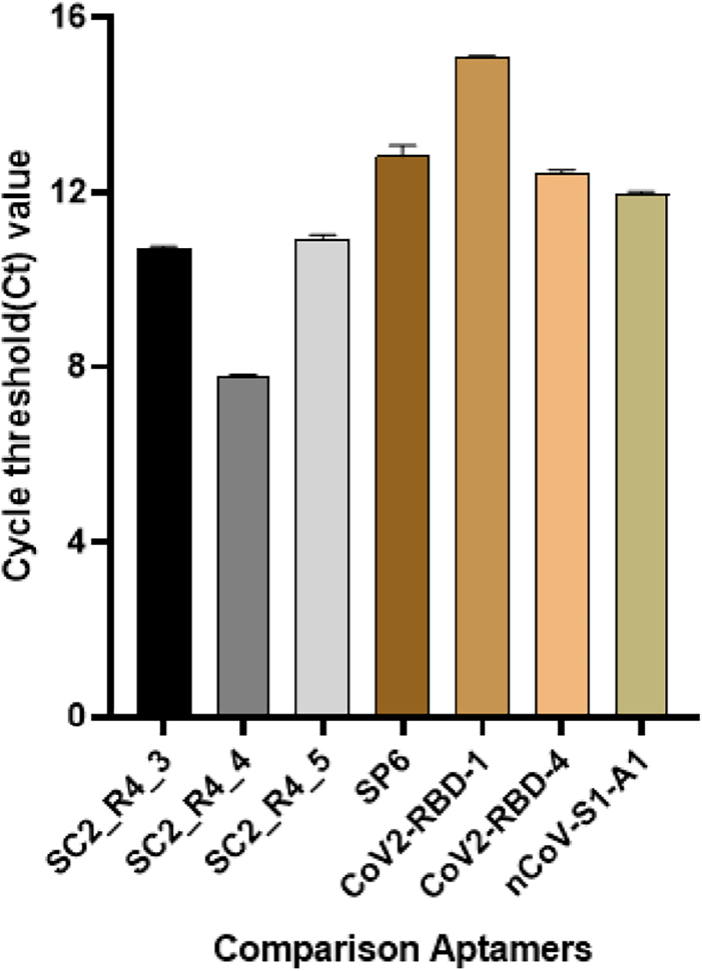
Comparison of binding affinity with other DNA aptamers. The Y-axis represents the Ct values, and each bar on the X-axis represents the comparison between the selected DNA aptamers and the comparative DNA aptamers. The experiment was conducted using the same method as before. Error bars indicate the standard deviation obtained from three parallel tests.

In addition, the SELEX method and qPCR were employed following the same protocol as in this study to compare the binding affinities of the DNA aptamers developed in other studies (Table 2). A comparison between the three candidate DNA aptamers identified in this study and four types of DNA aptamers from previous studies, showed that all candidate aptamers exhibited higher binding affinities (Fig. 3). These results indicate that the DNA aptamer developed in our study has a higher binding affinity to the SARS-CoV-2 spike protein.

**Table 2:** List of recently reported DNA aptamers.

No	Name	5′-Oligo Seq-3′	References
1	nCoV-S1-A1	AGC AGC ACA GAG GTC AGA TGC CGC AGG CAG CTG CCA TTA GTC TCT ATC CGT GAC GGT ATG CCT ATG CGT GCT ACC GTG AA	^20^
2	SP6	GGG AGA GGA GGG AGA TAG ATA TCA ACC CAT GGT AGG TAT TGC TTG GTA GGG ATA GTG GGC TTG ATG TTT CGT GGA TGC CAC AGG AC	^45^
3	CoV2-RBD-1	ATC CAG AGT GAC GCA GCA CCG ACC TTG TGC TTT GGG AGT GCT GGT CCA AGG GCG TTA ATG GAC ACG GTG GCT TAG T	^44^
4	CoV2-RBD-4	ATC CAG AGT GAC GCA GCA TTT CAT CGG GTC CAA AAG GGG CTG CTC GGG ATT GCG GAT ATG GAC ACG GTG GCT TAG T	^44^

### Secondary structures of candidate DNA aptamers

**Figure 4:**
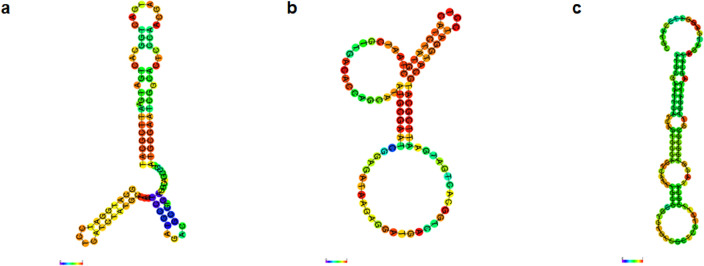
The minimum free energy (MFE) secondary structure of DNA aptamers predicted by the RNAfold web server. The structural predictions were generated using the RNAfold server from the Vienna websuit (http://rna.tbi.univie.ac.at). The predicted secondary structures of (**a**) SC2_R4_3, (**b**) SC2_R4_4, and (**c**) SC2_R4_5 exhibited stable loop-stem and bulge structures. The base pair probabilities calculated by the web server are color-coded, with blue representing the lowest probability (0) and red representing the highest probability (1).

The secondary structures of the three DNA aptamers with the highest binding affinities were predicted using the RNAfold web server based on the principle of minimum folding energy. The predicted secondary structures of these DNA aptamers displayed stable loop-stem and bulge structures. This suggests that the stem loop and bulge structures likely play significant roles in the binding of DNA aptamers to the SARS-CoV-2 spike protein. Figure 4 illustrates the predicted secondary structures of aptamers SC2_R4_3, SC2_R4_4, and SC2_R4_5, along with their respective minimum free energies (ΔG) of − 14.86, − 11.78, and − 12.17 kcal/mol.

### DNA aptamers suppress pro-inflammatory responses by interfering with the spike protein/ACE2 interaction

Numerous studies have demonstrated that proinflammatory cytokine levels increase during SARS-CoV-2 infection^34–36^. It is also well-known that the interaction between the ACE2 receptor and the spike protein of SARS-CoV-2 leads to a pro-inflammatory response^37–42^. Therefore, we aimed to confirm whether the developed DNA aptamer targeting the SARS-CoV-2 spike protein could reduce the pro-inflammatory response by interfering with the spike protein/hACE2 interaction. Consequently, we investigated the effect of the selected aptamer on pro-inflammatory signaling pathways.

**Figure 5:**
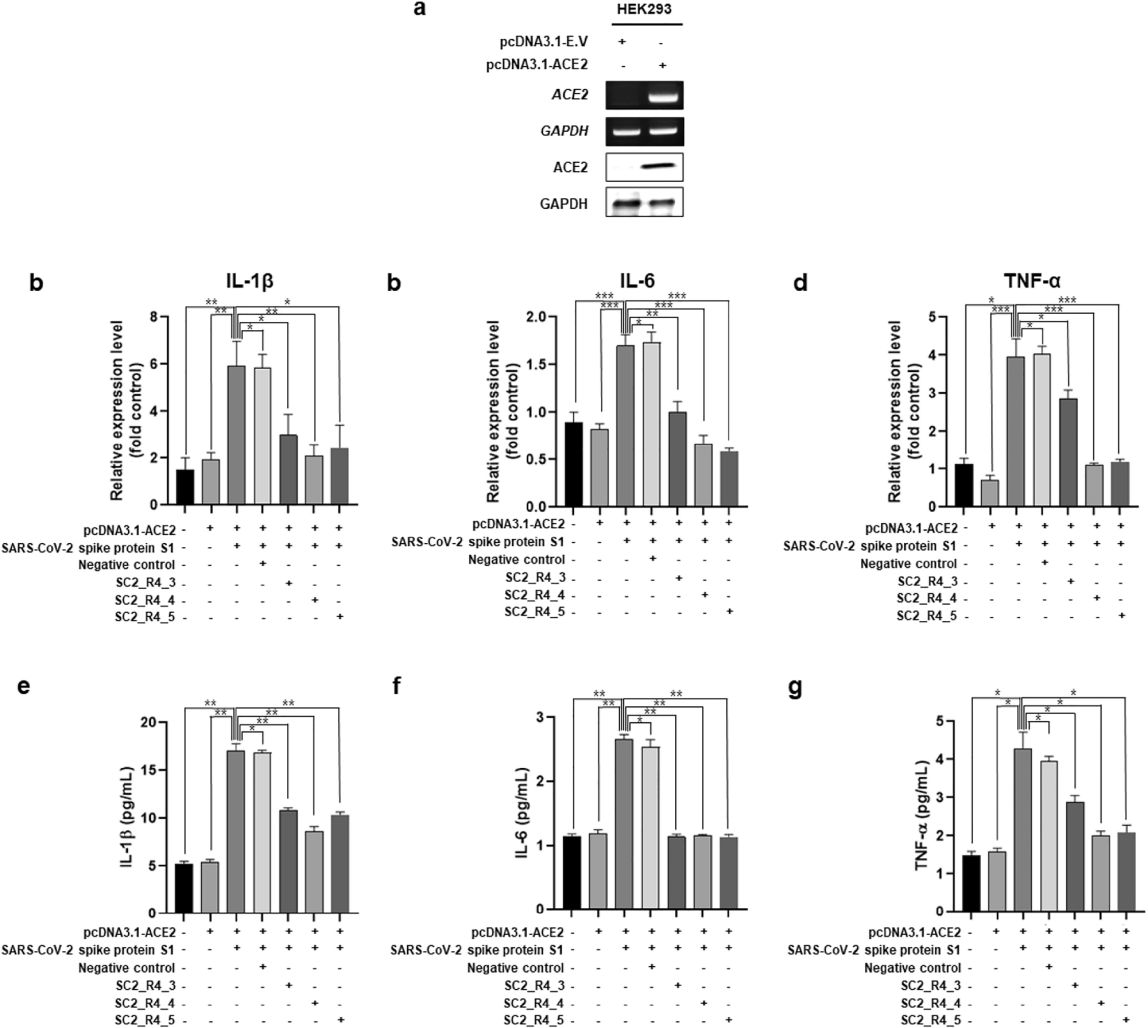
Inhibitory effect of DNA aptamers on the pro-inflammatory response induced by SARS-CoV-2 spike protein in HEK293 cells. (**a**) HEK293 cells were transfected with pcDNA3.1-E.V or pcDNA3.1-ACE2 over-expression levels were detected by RT-PCR and WB. GAPDH was used positive control. (**b**–**d**) To confirm the inhibition of the pro-inflammatory response of the candidate aptamer, 0.4 µg/mL of SARS-CoV-2 spike protein was bound with 1 µg/mL of DNA aptamers at 4 °C on a rotator for 30 min. The binders were then treated for 24 h in ACE2-overexpressing HEK293 cells. After incubation, mRNA expression of IL-1β (**b**), IL-6 (**c**), and TNF-α (**d**) were confirmed using qPCR, and cytokine levels [IL-1β (**e**), IL-6 (**f**), and TNF-α (**g**)] of culture medium was measured using ELISA kit. Negative control is random sequence ssDNA aptamer. The bar graph shows mRNA expression normalized to GAPDH (**b**,**c**,**d**) and cytokine concentration (**e**,**f**,**g**). (*, **, ***, and ns indicate *P* < 0.1, *P* < 0.01, *P* < 0.001, and not significant, respectively).

To enhance the response to the SARS-CoV-2 spike protein, we overexpressed ACE2 in HEK293 cells. HEK293 cells were transiently transfected with the pcDNA3.1-E. V or pcDNA3.1-ACE2, and the overexpression of ACE2 was confirmed by RT-PCR and WB (Fig. 5a). Subsequently, ACE2-overexpressing HEK293 cells were treated with the selected DNA aptamers and SARS-CoV-2 spike proteins. As a result, we observed a decrease in the expression levels of pro-inflammatory cytokines Il-1β, IL-6, and TNF-α upon treatment with all types of DNA aptamers (Fig. 5b–d). In addition, cytokine levels decreased with the DNA aptamers (Fig. 5e–g). The reduction in cytokine levels exhibited a similar pattern to that of the binding affinity of the DNA aptamers to the SARS-CoV-2 spike protein (Fig. 5b–g). These findings suggest that the developed DNA aptamer inhibits the interaction between the SARS-CoV-2 spike protein and ACE2 by binding to them.

## Discussion

The SARS-CoV-2 spike protein on the viral envelope can bind to ACE2 via the RBD in the S1 subunit^6^. Compared to RNA aptamers, DNA aptamers are more stable and therefore easier to chemically modify and immobilize^43^. In this study, nine candidate DNA aptamers that bind to the SARS-CoV-2 spike protein were developed using the SELEX method. The binding affinities of the selected DNA aptamers were compared with the Ct values obtained using qPCR. The best aptamers for SARS-CoV-2 spike protein binding identified in this study were SC2_R4_3, SC2_R4_4, and SC2_R4_5. In addition, the superiority of our aptamers was confirmed by comparing their binding affinities with those of aptamers developed in previous studies^20,44,45^. These results showed that the affinity of our selected aptamer for the SARS-CoV-2 spike protein was higher than that of the other comparative DNA aptamers.

To gather structural information on candidate aptamers, their secondary structures were predicted by the RNAfold Webserver using DNA parameters^27^. The predicted secondary structures of three selected aptamers are shown in Fig. 4. The minimum free energy (MFE) predicted the secondary structures of the aptamers, including loop-stem and bulge structures.

Finally, we investigated the effect of the selected aptamer on cellular responses through molecular experiments. Recent studies suggest that an excessive pro-inflammatory response in the host drives the disease severity and mortality in patients^35,36^. In addition, the proinflammatory factors TNF-α, IL-1β, and IL-6 have been suggested as biomarkers of SARS-CoV-2 infection^34^. Thus, we confirmed that the developed DNA aptamers reduced the proinflammatory response by interfering with the spike protein/ACE2 interaction. These findings indicated that all types of DNA aptamers successfully decreased the levels of pro-inflammatory cytokines.

In this study, a DNA aptamer that binds to the SARS-CoV-2 spike protein was developed; however, direct binding between the aptamer and protein could not be confirmed. Since various studies have recently been conducted to measure the binding affinity of aptamers using qPCR^30–33^, we were able to indirectly confirm the binding between the aptamer and protein by measuring the Ct value. In addition, indirect binding between the DNA aptamer and SARS-CoV-2 spike protein was confirmed by utilizing the pro-inflammatory response caused by surface interaction between the ACE2 receptor and SARS-CoV-2 spike protein^37–42^. As expected, the decrease in cytokine levels showed a pattern similar to that of the binding affinity of DNA aptamers for the SARS-CoV-2 spike protein. As a result, we could not confirm the molecular mechanism by which SC2_R4_3, SC2_R4_4, and SC2_R4_5 inhibited viral infection, but we could indirectly confirm that the DNA aptamer interfered with the binding of the SARS-CoV-2 spike protein to ACE2.

In conclusion, we developed DNA aptamers that bind to the SARS-CoV-2 spike protein and have the potential to inhibit SARS-CoV-2 infection. The three selected DNA aptamers had higher affinities than DNA aptamers developed in other studies. Furthermore, we confirmed that the developed DNA aptamer reduced the pro-inflammatory response by interfering with the spike protein/ACE2 interaction. In conclusion, DNA aptamers can be used to inhibit SARS-CoV-2 infection, and this study intends to provide a direction for the development of new therapeutics.

### Supplementary Information

Supplementary Information.

## Data Availability

The datasets generated and/or analysed during the current study are not publicly available due the confidentiality reasons, but are available from the corresponding author on reasonable request.
