# Supplementary figures and images for: A new DNA aptamer which binds to SARS-CoV-2 spike protein and reduces pro-inflammatory response
Source: Sci Rep. 2024 Mar 29;14:7516. doi: 10.1038/s41598-024-58315-0 (PMC10980804; doi:10.1038/s41598-024-58315-0)

Fig 5.A

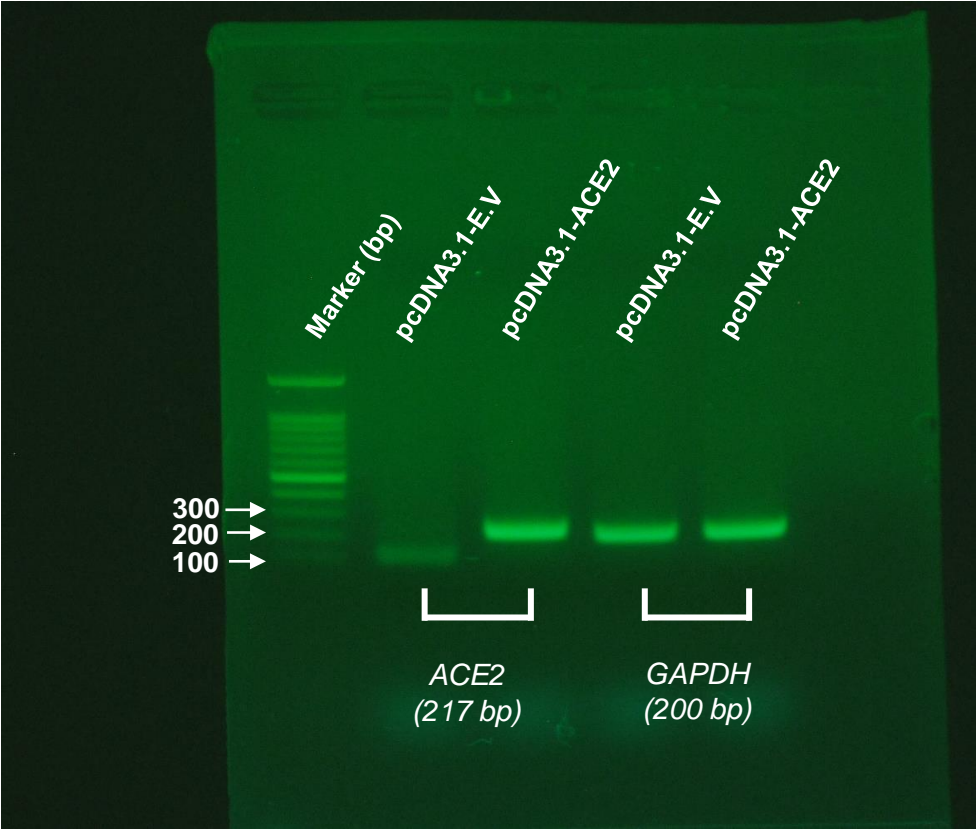

Fig 5.A

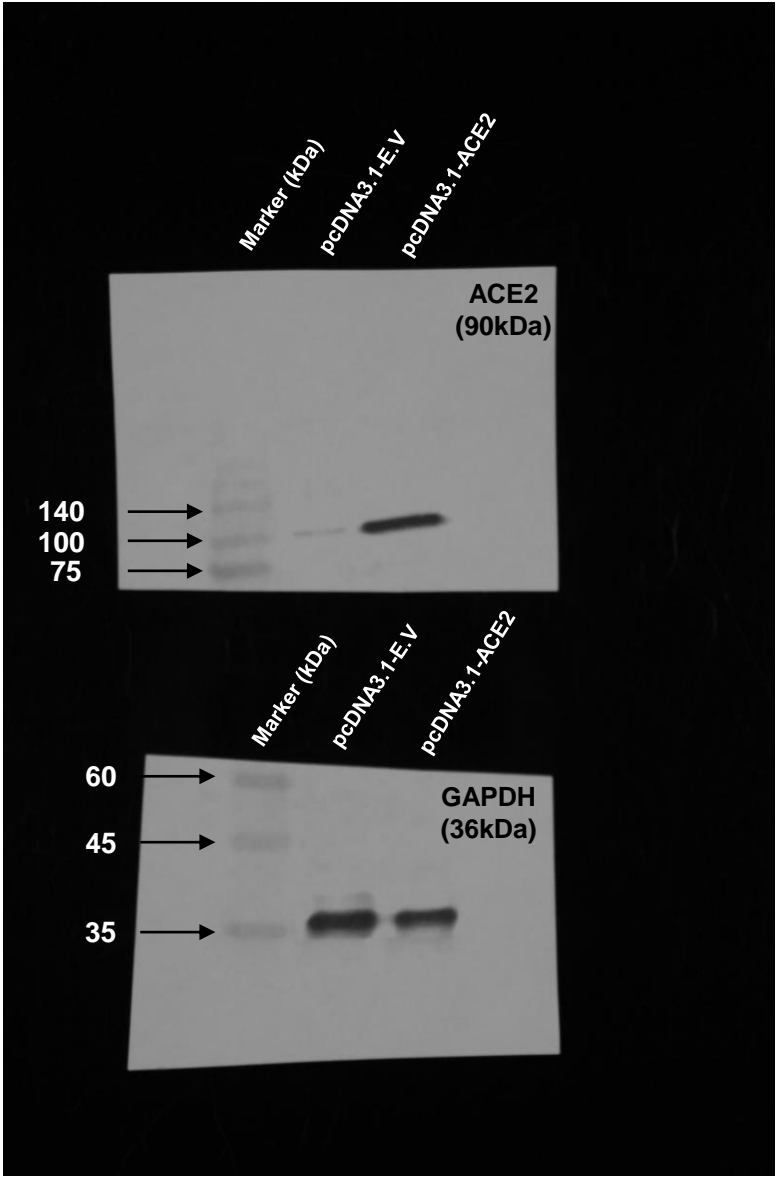

Supplement: Supplementary file 1 — Supplementary Information. [file 41598_2024_58315_MOESM1_ESM.pdf]
